# A novel nomogram to predict the risk of anastomotic leakage in patients after oesophagectomy

**DOI:** 10.1186/s12893-020-00726-7

**Published:** 2020-04-06

**Authors:** Chengya Huang, Haixia Yao, Qi Huang, Huijie Lu, Meiying Xu, Jingxiang Wu

**Affiliations:** Department of Anaesthesiology, Shanghai Chest Hospital, Shanghai Jiao Tong University, No. 241 Huaihai Rd. West, Shanghai, China

**Keywords:** Oesophagus operation, Anastomotic leakage, Predictors, Nomogram

## Abstract

**Background:**

Anastomotic leakage is a dangerous postoperative complication of oesophageal surgery. The present study aimed to develop a simple and practical scoring system to predict the risk of anastomotic leakage after oesophageal resection.

**Methods:**

A consecutive series of 330 patients who underwent oesophageal cancer surgery from January 2016 to January 2018 at the Shanghai Chest Hospital were included to develop a prediction model. Anastomotic leakage was evaluated using oesophagography, computed tomography, or flexible endoscopy. Least absolute shrinkage and selection operator regression based on a generalized linear model was used to select variables for the anastomotic leakage risk model while avoiding overfitting. Multivariable logistic regression analysis was applied to build forest plots and a prediction model. The concordance index or the area under the curve was used to judge the discrimination, and calibration plots verified the consistency. Internal validation of the model was conducted, and the clinical usefulness and threshold screening of the model were evaluated by decision curve analysis.

**Results:**

The factors included in the predictive nomogram included Sex, diabetes history, anastomotic type, reconstruction route, smoking history, CRP level and presence of cardiac arrhythmia. The model displayed a discrimination performance with a concordance index of 0.690 (95% confidence interval: 0.620–0.760) and good calibration. A concordance index value of 0.664 was maintained during the internal validation. The calibration curve showed good agreement between the actual observations and the predicted results.

**Conclusion:**

The present prediction model, which requires only seven variables and includes Sex, diabetes history, anastomotic type, reconstruction route, smoking history, CRP level and presence of cardiac arrhythmia, may be useful for predicting anastomotic leakage in patients after oesophagectomy.

## Background

Anastomotic leakage (AL) is one of the most common serious complications after oesophageal surgery and causes prolonged hospital stay, intensive care unit (ICU) admission, and even mortality [1–5]. The incidence of AL is approximately 6–24% in patients undergoing oesophagogastrostomy [2, 6], with an increased postoperative mortality rate of 7.2–18.2% compared with that of 3.1–6.2% in patients without leakage. The cost burden was two-times higher for high-grade leakages in an analysis of cervical oesophagogastric anastomotic leaks after transhiatal oesophagectomy [7]. The early detection of AL is crucial since delayed treatment is associated with significant morbidity, a prolonged hospital stay, and mortality. The common risk factors for postoperative AL are generally believed to be Sex, age, and the general condition of the patient (long-term malnutrition in patients with oesophageal cancer and a state of negative nitrogen balance, which is often accompanied by chronic wasting diseases such as anaemia and hypoproteinaemia that arise from the body’s poor tolerance to surgery). Moreover, the anastomotic type, anastomotic approach, anastomotic site, anastomotic tension, blood supply to the anastomotic region, intrathoracic infection, and postoperative digestive tract emptying are also related to AL [8]. Since there are many relevant risk factors, the use of accurate predictive tools and early intervention may be the most effective preventive actions for postoperative AL. Although relevant guidelines for this research area exist both internationally and domestically, there is a lack of validated tools based on national characteristics. This study aimed to develop a simple and effective prediction tool to perioperatively estimate the risk of AL for oesophageal cancer patients using readily available characteristics at the start of therapy.

## Methods

### Patients and data collection

This retrospective cohort study was approved by the institutional review board of Shanghai Chest Hospital and Shanghai Jiao Tong University’s Ethics Committee, and the requirement to obtain informed consent was waived. A total of 330 patients underwent three-incision oesophageal surgery. The data were collected from January 2016 to January 2018. The demographic characteristics, clinical features and auxiliary examinations of the patients were recorded in detail.

### Protocol

According to the reference literature and relevant clinical experience, a total of 45 variables with potential connections to AL were included [9, 10]. The study was completed using an independent database from the hospital, and the database was automatically updated. At our centre, if AL was first suspected on the basis of factors such as persistent fever, leucocytosis, unexplained pneumonia, abnormal chest tube drainage that included faecal contents, and local inflammation of a cervical wound, then the leak could be visualized by contrast oesophagography, computed tomography, or flexible endoscopy [11].

The following variables were recorded:

(i) Clinical examination: Sex, age at surgery, body mass index (BMI), cardiac ultrasound, liver and kidney function, blood biochemistry, cardiovascular risk factors (smoking history (at least 10 cigarettes a day for more than 5 years), diabetes mellitus (DM) history (diagnosed as type 2 DM for at least one year before surgery according to the WHO criteria and receiving oral antidiabetic drugs or insulin), chronic hypertension and coronary heart disease (diagnosed by a physician and currently taking antihypertensive, antiplatelet or antianginal drugs or having a history of myocardial revascularization), location of oesophageal tumours and preoperative neoadjuvant chemoradiotherapy (nCRT).
(ii) Perioperative data: surgical method, duration of the procedure, American Society of Anesthesiologists (ASA) score, anastomotic type, reconstruction route, duration of anaesthesia, intubation method, oxygen saturation level, temperature, hypotension status, hypertension status, presence of cardiac arrhythmia (new-onset intraoperative cardiac arrhythmia, such as atrial fibrillation, ventricular tachycardia, frequent multisource ventricular premature beats and severe paroxysmal supraventricular tachycardia that needed drug or electrical conversion therapy) and the change in central venous pressure when entering the thoracic cavity.

Disease stages and tumour types were not included in the variable screening for the model, although they were reported to be important risk factors. However, in clinical practice, the results of the pathological tumour-metastasis-node (TMN) classification of tumours are reported relatively late. Considering that this study mainly focused on the early prediction capabilities of the model, the parameters selected were mainly preoperative and intraoperative indicators.

### Operations

Our standard procedures consisted of a three-incision surgery (thoracic cavity, abdominal stations and cervical), reconstruction with a gastric tube through a posterior mediastinal route or retrosternal route, and anastomosis in the cervical incision. The thoracic phase consisted of either thoracotomy or video-assisted or robot-assisted thoracoscopic surgery. Lymph node dissection was based on total two-field lymphadenectomy, which included (1) all nodes and periesophageal tissues below the level of the carina to the celiac trifurcation and (2) all superior mediastinal nodes along the recurrent laryngeal nerve to the lower poles of the thyroid. Lymph nodes in the supraclavicular fossa were not routinely removed. The abdominal phase was performed using laparotomy or laparoscopy. Patients received either a hand-sewn or circular-stapled cervical oesophagogastric anastomosis.

### Statistical analysis

In the first step, the least absolute shrinkage and selection operator (LASSO) regression model, which is based on a generalized linear model, was used to identify suitable predictive features [12]. Second, multivariable logistic regression analysis was performed to build a forest plot and a prediction model. The model that included all candidate predictors selected from the LASSO analysis was presented as a nomogram. Third, we validated the discrimination of the nomogram with Harrell’s concordance index (C-index) and the area under the curve (AUC). To assess the calibration of the nomogram, bias-corrected calibration plots were used to compare the actual risk and predicted risk. Meanwhile, decision curve analysis (DCA) was used to estimate the clinical usefulness and net benefit of the nomogram [13]. Finally, the nomogram was internally validated using 1000 bootstrap resamples and calculating a relatively corrected C-index.

All *P*-values less than 0.05 were considered statistically significant, and the statistical significance levels were two-sided. In this study, the data were analysed with R software (Version 3.5.2; https://www.R-project.org). The packages included glmnet, rms, ROCR, rmda and forestplot.

## Results

### Patient characteristics

Baseline characteristics, including demographics, disease, and treatment characteristics of the patients, are summarized in Table 1. A total of 330 patients (263 males and 67 females; mean age 62.83 ± 7.22 years [range 42–83 years]) who underwent oesophageal surgery were enrolled from January 2016 to January 2018. Among all the patients, 97.3% (321/330) had oesophageal squamous cell carcinoma, and 2.7% (9/330) had adenocarcinoma. Oesophagectomies were performed by 10 specialist surgeons. The surgical method was jointly decided after discussion by the surgical team, and standard operating procedures were used. Patients were divided into an AL group and a non-AL group.

**Table 1:** Differences between demographic and clinical characteristics of AL and non-AL groups

Characteristics	AL	Non- AL	Population
	(*n* = 79, n%)	(*n* = 251, n%)	(*n* = 330, n%)
**Sex**			
male	68 (86.08)	195 (77.69)	263 (79.70)
female	11 (13.92)	56 (22.31)	67 (20.30)
**Age (years)**			
< 65	49 (62.03)	144 (57.37)	193 (58.48)
≥ 65 and < 75	27 (34.18)	93 (37.05)	120 (36.36)
≥ 75	3 (3.80)	14 (5.58)	17 (5.15)
**Pathology**			
squamous	78 (98.73)	243 (96.81)	321 (97.27)
adenocarcinoma	1 (1.27)	8 (3.19)	9 (2.73)
**Pathologic tumour stage**			
Stage 0	0 (0)	0 (0)	0 (0)
Stage I	9 (11.39)	51 (20.32)	60 (18.18)
Stage II	36 (45.57)	109 (43.43)	145 (43.94)
Stage III	32 (40.51)	86 (34.26)	118 (35.76)
Stage IV	2 (2.53)	5 (1.99)	7 (2.12)
**Surgery method**			
robot	27 (34.18)	84 (33.47)	111 (33.64)
thoracoscopy	31 (39.24)	122 (48.61)	153 (46.36)
open	21 (26.58)	45 (17.93)	66 (20)
**HBP**	7 (8.86)	21 (8.37)	28 (8.48)
**DM**	12 (15.19)	20 (7.97)	32 (9.70)
**CHD**	3 (3.80)	3 (1.20)	6 (1.82)
**Smoking**	12 (15.19)	14 (5.58)	26 (7.88)
**nCRT**	13 (16.46)	28 (11.16)	41 (12.42)
**Location**			
upper	10 (12.66)	32 (12.75)	42 (12.73)
middle	47 (59.49)	163 (64.94)	210 (63.64)
lower	22 (27.85)	56 (22.31)	78 (23.64)
**BMI (kg/m**^**2**^**)**			
< 18.5	7 (8.86)	16 (6.37)	23 (6.97)
≥ 18.5 and <24	39 (49.37)	148 (58.96)	187 (56.67)
≥ 24 and <28	27 (34.18)	77 (30.68)	104 (31.52)
≥ 28	6 (7.59)	10 (3.98)	16 (4.85)
**ASA**			
I	1 (1.27)	5 (1.99)	6 (1.82)
II	69 (87.34)	222 (88.45)	291 (88.18)
III	9 (11.39)	24 (9.56)	33 (10)
**Blood (≥400 ml)**	3 (3.80)	11 (4.38)	14 (4.24)
**Surgery time (min)**			
< 180	2 (2.53)	12 (4.78)	14 (4.24)
≥ 180 and 300	50 (63.29)	174 (69.32)	224 (67.88)
≥ 300	27 (34.18)	65 (25.90)	92 (27.88)
**Thoracic surgery time**			
< 60	9 (11.39)	25 (9.96)	34 (10.30)
≥ 60 and < 120	49 (62.03)	168 (66.93)	217 (65.76)
≥ 120	21 (26.58)	58 (23.11)	79 (23.94)
**Transfer**	6 (7.59)	10 (3.98)	16 (4.85)
**Anastomotic type**			
stapled	44 (55.70)	186 (74.10)	230 (69.70)
hand-sewn	35 (44.30)	65 (25.90)	100 (30.30)
**Route reconstruction**			
retrosternal	44 (55.70)	80 (31.87)	124 (37.58)
posterior mediastinum	35 (44.30)	171 (68.13)	206 (62.42)
**Cardiac ultrasound**			
abnormal	23 (29.11)	68 (27.09)	91 (27.58)
**TP (g/L)**			
< 65	8 (10.13)	18 (7.17)	26 (7.88)
≥ 65	71 (89.87)	233 (92.83)	304 (92.12)
**ALB (g/L)**			
< 35	5 (6.33)	7 (2.79)	12 (3.64)
≥ 35	74 (93.67)	244 (97.21)	318 (96.36)
**PAB (g/L)**			
< 0.2	11 (13.92)	38 (15.14)	49 (14.85)
≥ 0.2	68 (86.08)	213 (84.86)	281 (85.15)
**ALT (U/L)**			
< 9	3 (3.80)	16 (6.37)	19 (5.76)
≥ 9 and < 50	74 (93.67)	228 (90.84)	302 (91.52)
≥ 50	2 (2.53)	7 (2.79)	9 (2.73)
**AST (U/L)**			
< 15	4 (5.06)	22 (8.76)	26 (7.88)
≥ 15 and < 40	72 (91.1)	210 (83.67)	282 (85.45)
≥ 40	3 (3.80)	19 (7.57)	22 (6.67)
**Urea (mmol/L)**			
< 3.1	1 (1.27)	5 (1.99)	6 (1.82)
≥ 3.1 and < 9.5	77 (97.47)	243 (96.81)	320 (96.97)
≥ 9.5	1 (1.27)	4 (1.59)	5 (1.52)
**CREA (mmol/L)**			
< 57	1 (1.27)	5 (1.99)	6 (1.82)
≥ 57 and < 111	76 (96.20)	240 (95.62)	316 (95.76)
≥ 111	2 (2.53)	6 (2.39)	8 (2.42)
**Glu (mmol/L)**			
< 4.3	6 (7.59)	13 (5.18)	19 (5.76)
≥ 4.3 and < 5.9	57 (72.15)	190 (75.70)	247 (74.85)
≥ 5.9	16 (20.25)	48 (19.12)	64 (19.39)
**PCT (ng/ml)**			
0–0.05	67 (84.81)	219 (87.25)	286 (86.67)
> 0.05	12 (15.19)	32 (12.75)	44 (13.33)
**D-dimer (mg/L)**			
< 0.55	64 (81.01)	195 (77.69)	259 (78.48)
≥ 0.55	15 (18.99)	56 (22.31)	71 (21.52)
**CRP (mg/L)**			
≤ 10	70 (88.61)	238 (94.82)	308 (93.33)
> 10	9 (11.39)	13 (5.18)	22 (6.67)
**ESR (mm/h)**			
≤ 40	62 (78.48)	198 (78.88)	260 (78.79)
> 40	17 (21.52)	53 (21.11)	70 (21.21)
**PLT (10^9/L)**			
< 125	8 (10.13)	16 (6.37)	24 (7.27)
≥ 125 and < 350	69 (87.34)	224 (89.24)	293 (88.79)
≥ 350	2 (2.53)	11 (4.38)	13 (3.94)
**Hb (g/L)**			
< 130	17 (21.52)	47 (18.73)	64 (19.39)
≥ 130 and < 175	61 (77.22)	200 (79.68)	261 (79.09)
≥ 175	1 (1.27)	4 (1.59)	5 (1.52)
**HCT (%)**			
< 40	18 (22.78)	43 (17.13)	61 (18.48)
≥ 40 and < 50	56 (70.89)	191 (76.10)	247 (74.85)
≥ 50	5 (6.33)	17 (6.77)	22 (6.67)
**Anaesthesia time (min)**			
< 300	31 (39.24)	99 (39.44)	130 (39.39)
≥ 300 and < 420	40 (50.63)	143 (56.97)	183 (55.45)
≥ 420	8 (10.13)	9 (3.59)	17 (5.15)
**Anaesthesia method**			
GA	75 (94.94)	238 (94.82)	313 (94.85)
GA + NB	4 (5.06)	13 (5.18)	17 (5.15)
**Intubation**			
DLT	34 (43.04)	79 (31.47)	113 (34.24)
Tracheal + BBT	17 (21.52)	79 (31.47)	96 (29.09)
Tracheal tube	28 (35.44)	93 (37.05)	121 (36.67)
**SPO**_**2**_**(%)**			
< 90	68 (86.08)	220 (87.65)	288 (87.27)
≥ 90	11 (13.92)	31 (12.35)	42 (12.73)
**EtCO**_**2**_**_max (mmHg)**			
< 60	77 (97.47)	239 (95.22)	316 (95.76)
≥ 60	2 (2.53)	12 (4.78)	14 (4.24)
**PaCO**_**2**_**(mmHg)**			
< 65	64 (81.01)	194 (77.29)	258 (78.18)
≥ 65	15 (18.99)	57 (22.71)	72 (21.82)
**Protective ventilation**			
no	47 (59.49)	143 (56.97)	190 (57.58)
yes	32 (40.51)	108 (43.03)	140 (42.42)
**Temperature (°C)**			
< 36.0	55 (69.62)	173 (68.92)	228 (69.09)
≥ 36.0	24 (30.38)	78 (31.08)	102 (30.91)
**Hypotension**			
no	73 (92.41)	232 (92.43)	305 (92.42)
< SBP 90 mmHg, ≥10 min	5 (6.33)	17 (6.77)	22 (6.67)
> SBP 90 mmHg, ≥30 min	1 (1.27)	2 (0.80)	3 (0.91)
**Hypertension**			
no	63 (79.75)	199 (79.28)	262 (79.39)
> SBP160 mmHg, ≥10 min	10 (12.66)	42 (16.73)	52 (15.76)
> SBP160 mmHg, ≥30 min	6 (1.82)	10 (3.98)	16 (4.85)
**Cardiac arrhythmia**			
no	69 (87.34)	240 (95.62)	309 (93.64)
yes	10 (12.66)	11 (4.38)	21 (6.36)
**CVP change (cmH**_**2**_**O)**			
0–5	9 (11.39)	30 (11.95)	39 (11.82)
5–10	35 (44.30)	104 (41.43)	139 (42.12)
10–15	28 (35.44)	90 (35.86)	118 (35.76)
≥ 15	7 (8.86)	27 (10.76)	34 (10.30)

*Abbreviations*: *HBP* hypertension, *DM* diabetes, *CHD* coronary heart disease, *TP* total protein, *ALB* albumin, *PAB* prealbumin, *ALT* alanine aminotransferase, *AST* aspartate transaminase, *PCT* procalcitonin, *CRP* C-reactive protein, *ESR* erythrocyte sedimentation rate, *PLT* platelet count, *Hb* haemoglobin, *HCT* red blood cell specific volume, *CVP* central venous pressure, *nCRT* neoadjuvant chemoradiotherapy

### Variable selection

**Fig. 1:**
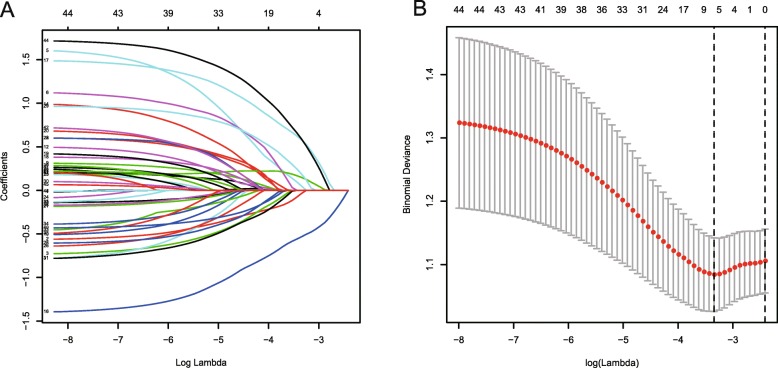
Predictor selection using a LASSO logistic regression model. **a** Shrinkage estimation parameter: fivefold cross validation (CV) is the most common repeated sampling method. According to the one standard error (SE) rule, glmnet usually recommends lambda.1se instead of lambda.min to identify the model with the best performance but with the fewest number of independent variables. **b** Dotted vertical lines were drawn at the optimal values by using the minimum criteria (lambda.min) and 1 SE of the minimum criteria (lambda.1se)

LASSO regression analysis can be used to reduce the dimensionality of complex variables and increase the accuracy of a model. Finally, baseline characteristics that included 45 features were reduced to 7 potential predictors (Fig. 1). The strongest predictors included Sex, DM history, anastomotic type, reconstruction route, smoking history, CRP level and presence of cardiac arrhythmia (Table 2).

**Table 2:** Prediction factors for the risk of anastomotic leakage with oesophageal operation

Intercept and variable	β	Prediction model	*P*-value
		Odds ratio (95% CI)	
Intercept	−1.05	0.35 (0.19–0.63)	0.001 ***
Sex (female)	−0.58	0.56 (0.26–1.14)	0.13
DM	0.57	1.80 (0.73–4.24)	0.19
Anastomotic type (hand-sewn)	0.39	1.47 (0.78–2.75)	0.23
Reconstruction route (mediastinal)	−0.87	0.42 (0.23–0.77)	0.004 **
Smoking	1.12	3.08 (1.24–7.50)	0.01 *
CRP (> 10 mg/L)	0.83	2.28 (0.85–5.92)	0.09
Cardiac arrhythmia	1.42	4.12 (1.57–10.72)	0.003 **

*P<0.05, **P<0.01, ***P<0.001

### Development of a novel prediction model

**Fig. 2:**
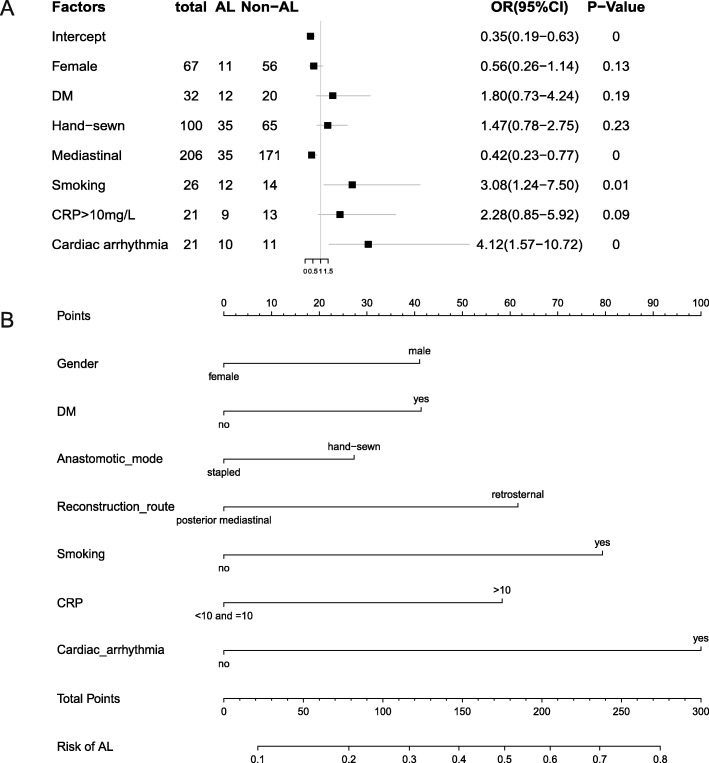
**a** Multivariable logistic regression analysis was applied to build the forest plots. **b** The developed AL risk nomogram. Note: The AL risk nomogram was developed by incorporating the following characteristics: sex, DM history, anastomotic type, route reconstruction, smoking history, CRP level and presence of cardiac arrhythmia

The weights and points associated with the seven variables are shown in the forest plots (Fig. 2a). Additionally, all potential predictors in Table 2 were used to develop a prediction model for the risk of AL and are presented as the nomogram (Fig. 2b).

### How to use this nomogram

As seen in Fig. 2b, for one patient, the corresponding point is derived from each variable axis. The sum of the points is located on the total points axis and corresponds to a probability shown below (risk of AL). For example, if a male smoker with a hand-sewn anastomosis undergoes surgery and he has a sudden new onset of atrial fibrillation, the total points would be 248, and the risk of AL would be 73%. This would help us to treat patients for AL and to arrange follow-up treatment.

### Evaluation of the model

**Fig. 3:**
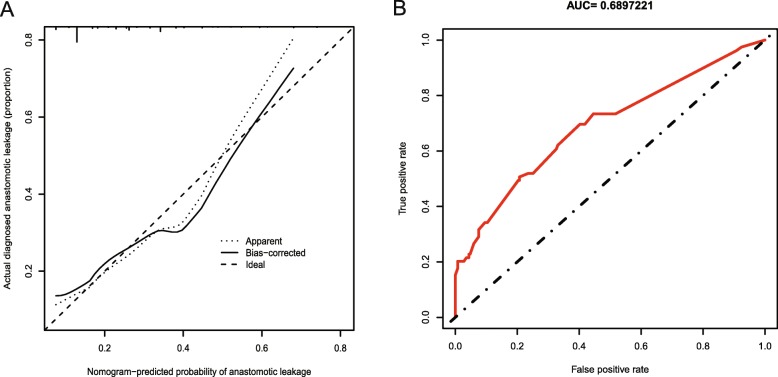
**a** Calibration plots of the nomogram. Notes: The y-axis is the actual rate of AL diagnosis. The x-axis is the predicted risk of AL. The diagonal dotted line represents a perfect prediction by an ideal model. The solid line represents the bias−corrected performance of the nomogram, where a closer fit to the diagonal dotted line represents a better prediction. **b** The accuracy of the model for identifying patients with AL was determined using AUC analysis

The predictive nomogram achieved a C-index of 0.690 (95% confidence interval (CI): 0.620–0.760), which was confirmed to be 0.664 through internal validation, suggesting that the model has moderate discrimination. A calibration curve was based on the actual incidence and predicted incidence. The bias-corrected line was the fitted line of the predicted and measured incidence. For convenience during comparisons, an ideal dotted line was added to the figure. The dotted line represents y = x, which means that the predicted and measured rates are exactly the same. The calibration curve of the nomogram to predict AL risk after oesophageal surgery demonstrated good agreement in this cohort (Fig. 3a). The accuracies of the risk models were also compared using area under the receiver operating characteristic (ROC) curve (AUC = 0.690) analysis (Fig. 3b).

### DCA for the prediction model

As previously described [14], DCA was used to determine whether the AL prediction model-based decisions were clinically useful compared with the default strategies for patients after oesophagectomy. DCA is based on a continuum of potential thresholds risk (x-axis, the harm-to-benefit ratio considering incorrect model-based decisions due to false negatives and false positives) and the net benefit of using the model to risk stratify patients (y-axis, potential benefit minus potential harm) relative to assuming that no patient will have AL. A model is only clinically useful at the threshold risk if it has a higher net benefit than treating all (slope line) and treating no (horizontal line) patients. If a model has a lower net benefit than any default strategy, we consider the model clinically harmful in that threshold risk range, as one of the default strategies leads to better decisions.

**Fig. 4:**
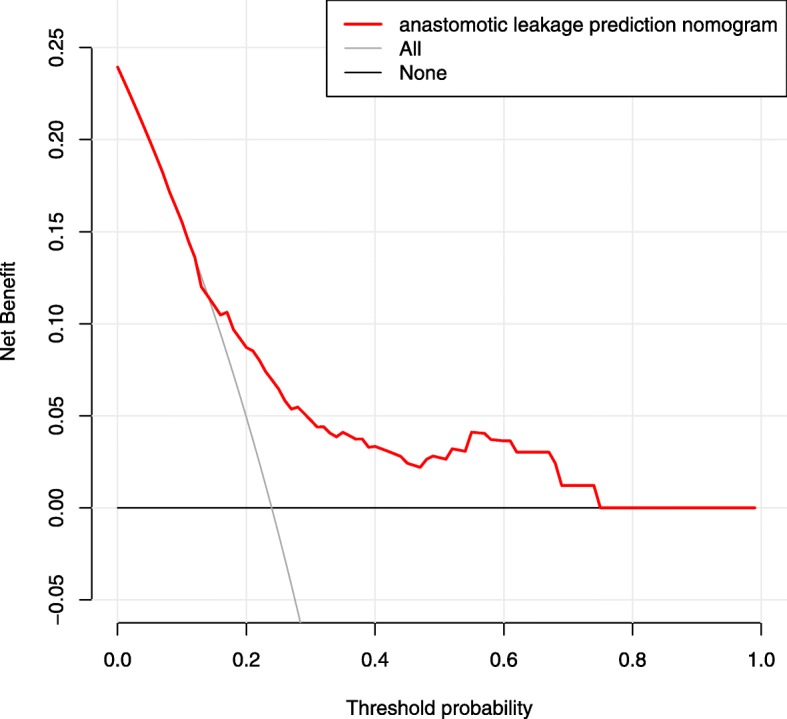
DCA. Notes: DCA showed the clinical usefulness of the nomogram. The y-axis measures the net benefit. The red solid line is the nomogram to predict AL risk. The grey solid line assumes that all patients will develop AL. The black thin solid line assumes that no patients will develop AL. In this analysis, the decision curve provided a larger net benefit across the range of 8 and 74%

As shown in Fig. 4, DCA graphically showed the clinical usefulness of the nomogram to predict AL risk. The graph shows the excepted net benefit per patient to predict the risk of AL for any patient (red curve). A typical range is 8–74%; that is, within this range of threshold risks, if an intervention based on AL predicts model-based decisions is clearly beneficial (e.g., delaying surgery to reduce the elevated CRP level), it should be used.

## Discussion

The current study developed a novel tool to predict the risk of AL after oesophageal surgery based on 2 years of data from indigenous Chinese patients. On the one hand, the prediction of the model focused on selecting predictors. When determining variables, the complexity of the model should be adjusted to achieve a balance between overfitting and insufficient fitting. LASSO regression analysis was used instead of univariate analysis to find independent risk factors. We believe that principal component analysis is only applicable to samples with few dimensions. LASSO is another data dimensionality reduction method, and it is suitable not only for linear data but also nonlinear data. LASSO can directly compress insignificant variable coefficients to 0 [15]. On the other hand, the accuracy and consistency of the model were evaluated in the trial, and the internal verification results showed that the nomogram can be widely used for AL prediction.

Two previous reports have made similar attempts to identify the risk of AL*.* One study was conducted by Haga Y et al. [16] in 2011, who created a prediction scoring system, “Estimation of Physiologic Ability and Surgical Stress” (E-PASS), to predict the postoperative morbidity and mortality of elective gastrointestinal surgery. The study included nine variables to calculate a numerical score. However, their research included various kinds of gastrointestinal surgery. There were only 292 oesophageal surgeries among the 6005 cases (4.8%), which may not fully reflect AL after oesophageal surgery. The other study was conducted in 2012 by Noble F et al. [17], who combined blood-borne inflammatory markers, including postoperative CRP levels, white cell counts and albumin levels, as predictors of AL and major complications. They reported a sensitivity of 95%, a specificity of 49% and a diagnostic accuracy of 0.801. The same nomogram was recently validated by Bundred J. et al., who reported an accuracy of 0.77 [18]. However, the three indicators used by Nobel F and Bundred J were laboratory indicators on the 4th day after surgery, which may be closely related to the anastomotic fistula itself. Their results can be used well for the treatment 4 days after surgery.

Previous studies have mainly focused on postoperative laboratory biomarker data, which is quite different from our study. The main advantage of the current study is that our nomogram is easier and more intuitive than existing methods, and our nomogram includes different parameters to consider both pre- and intraoperative factors. The current scoring tool is able to clarify the combination of surgical factors and patient factors by focusing on active smoking, CRP levels and intraoperative parameters, such as anastomotic type, reconstruction route and the presence of intraoperative cardiac arrhythmia. In addition, of the 45 empirical clinical parameters, 7 parameters with significant effects in the multivariate analysis were selected, and the weighting of each parameter was significant, which could reflect the significant influence of these factors on the predicted value.

In this study, approximately 24% of patients experienced AL after surgery. In a previous study, David T et al. [19] showed that a large number of preoperative comorbidities, advanced pathologic stage, postoperative arrhythmia, history of oesophagogastric surgeries, and active smoking history were risk factors for developing cervical oesophagogastric ALs, while a side-to-side stapled cervical oesophagogastric anastomosis was a protective factor [19]. A meta-analysis revealed that diabetes, preoperative serum albumin < 35 g/L, respiratory diseases, hypertension, preoperative neoadjuvant radiotherapy, stage III and IV oesophageal cancer, manual anastomosis and posterior sternal neck anastomosis were risk factors for AL after oesophageal cancer surgery [5].

We agree that patients with lower oesophageal carcinoma who received nCRT cannot be considered in the same way as patients treated for cervical cancer when analysing AL. However, we did not find a statistically significant difference based on the current data when including the factors of nCRT treatment and the location of the tumour (see Table 1). There is still some controversy about nCRT and AL. Although Briel et al. [20] reported that oesophagocolic anastomoses after nCRT were a risk factor for AL results from other studies did not support this finding [21–23].

Despite the fact that 37.2% of cases were T1-3N1-2M0 and T4aN0 − 1 M0, which were indications for nCRT according to the 2018 Chinese guidelines for diagnosis and treatment of oesophageal carcinoma [24], only 12.4% received nCRT because the use of nCRT before surgery in patients with locally advanced oesophageal cancer has only been slowly accepted in the last two years in China. Previously, it was believed that nCRT had a significant survival benefit for patients with locally advanced oesophageal cancer. However, nCRT was not recommended for patients with early oesophageal cancer because of its high surgical resection rate, and the addition of nCRT does not improve the R0 resection rate and increases the risk of postoperative death. In addition, even though nCRT appears to be associated with a higher R0 resection rate, there are still people who are reluctant to use preoperative nCRT because they are afraid of missing the appropriate surgical timing due to concerns about disease progression.

Urschel JD [25] and Pierie JP [26] suggested that an insufficient blood supply to the anastomotic stoma was the main risk factor for AL. Cooke found that postoperative arrhythmias that caused a low-flow state predicted leakage based on multivariate analysis [19]. Among our patients, those who experienced intraoperative arrhythmia were predisposed to AL, possibly for the same reason. The surgical approach is divided into a retrosternal reconstruction route and mediastinal reconstruction route. From an anatomical point of view, the length of the incision is longer with the posterior sternal approach than with the mediastinal approach, which inevitably leads to oesophageal free ends. An anastomotic route that is too long could cause excessive anastomotic tension, lack of blood supply to the tissue, and an elevated incidence of AL. Other potential risk factors, including smoking history and diabetic microangiopathy, in theory, may reduce microperfusion of tissues [19, 27]. The probability of postoperative AL is higher in men than in women; however, this difference is not clinically significant, which is consistent with the results from Vaporciyan AA [11]. The anastomotic techniques include hand-sewn anastomosis and device anastomosis. Hand-sewn anastomosis may cause variations in needle spacing and unequal ligation strength, which facilitates anastomotic stoma occurrence. CRP is an inflammatory indicator that can reflect the preoperative inflammatory condition of the body. High preoperative CRP levels may also increase the probability of developing AL by affecting tissue perfusion. Additionally, some studies have reported the utility of serum CRP levels for predicting postoperative inflammatory complications of oesophageal cancer before any clinical signs or symptoms appear [28–30].

Therefore, an accurate predictive model will help physicians assess the risk of AL and provide timely interventions. In actuality, it is difficult to predict AL in individual patients due to uncontrollable factors, such as the type of surgical procedure. However, early assessment, suitable examinations and multi-faceted interventions may still be the most effective method for preventing AL.

## Limitations

There are several limitations in our current study. First, the database was from a single, high-volume institute. Second, the study also has some limitations associated with its retrospective nature. Third, some prognostic parameters (such as pathological stage) and other important factors (such as drug history) were not included in our analysis. Finally, because external validation could not be conducted, we performed an internal validation using bootstrap testing.

## Conclusion

A novel risk score for the prediction of AL using Sex, diabetes history, anastomotic type, reconstruction route, smoking history, CRP level and the presence of cardiac arrhythmia was developed and internally validated. This nomogram offers a useful tool for clinicians to assess the risk of AL in individuals after surgery. The model focuses on preoperative and intraoperative data, which can be acquired earlier and more easily. Additionally, a prospective and international study is required to further validate the new nomogram.

## Data Availability

The datasets used and/or analysed during the current study are available from the corresponding author on reasonable request.

## Abbreviations

DM: Diabetes mellitus
ASA: American Society of Anesthesiologists
BMI: Body mass index
CRP: C-reactive protein
CVP: Central venous pressure
LASSO: Least absolute shrinkage and selection operator
SE: Standard error
